# Feasibility and safety study of the Flostent™ system (RAPID-I)

**DOI:** 10.1038/s41391-025-00984-7

**Published:** 2025-05-21

**Authors:** Dean Elterman, Ingrid Perscky, Ruben Urena, Alia Codelia-Anjum, Adam Kadlec, Bilal Chughtai

**Affiliations:** 1 Division of Urology, University of Toronto, Toronto, ON Canada; 2 Department of Urology, Pacífica Salud, Hospital Punta Pacífica, Panamá, Panama; 3 Department of Urology, Northwell Health, New York, NY USA; 4 Rivermark Medical, Inc., Milwaukee, WI USA

**Keywords:** Medical research, Prostatic diseases

## Abstract

**Introduction:**

This study aimed to evaluate the feasibility and safety of the FloStent™, a novel nitinol prostatic stent, in men with LUTS secondary to BPH.

**Materials and methods:**

This multi-center, single-arm, one-year study included men aged ≥45 with BPH. Participants underwent FloStent™ implantation using a flexible cystoscope. The primary endpoints were device implantability, tolerability, and device retrievability. Secondary assessments included International Prostate Symptom Score (IPSS), peak urinary flow rate (Qmax), post-void residual (PVR), and adverse events (AEs) at follow-ups over 52 weeks.

**Results:**

The study enrolled 15 participants with a mean age of 58.1 years. All attempts at implantation were successful. Postoperative catheterization was not required in any patient. Significant improvements were observed in IPSS and Qmax. Thirteen AEs were reported, all resolved, with no serious complications. Device retrieval was safely performed using standard urological equipment up to one-year post-implantation.

**Conclusions:**

This preliminary study indicates that the FloStent™ is a safe and effective treatment option for BPH, providing significant symptom relief and improved urinary function with a favorable risk profile. Further research, including larger, randomized controlled trials, are needed to validate these findings and establish the long-term efficacy and safety of the FloStent™ device.

## Introduction

Benign Prostatic Hyperplasia (BPH), the nonmalignant enlargement of the prostate gland, is a common condition with 94 million cases reported in 2019 in men over 40 years of age [1]. Most patients present with lower urinary tract symptoms (LUTS) secondary to BPH. There are multiple medical and surgical options for treatment of BPH. Medical therapy involves daily medication with one of three main pharmacological therapies: alpha-adrenergic sympathomimetic blocking agents, 5-alpha reductase inhibitors, or phosphodiesterase-5 inhibitors. Long-term use of BPH medication has been associated with sexual side effects, specifically erectile dysfunction and ejaculatory disorders [2–4]. While standard surgical treatments for BPH have provided significant and reliable improvement of LUTS, their associated risks and need for anesthesia can make some treatments unsuitable for certain patients [5, 6]. To address these concerns, first-line interventional therapy (FIT), uses minimally invasive surgical therapies (MISTs) to address the limitations of current treatments. MISTs provide the potential for BPH treatment without the safety risks associated with traditional surgical methods or the side-effects associated with medication [7, 8].

Rivermark Medical’s (Milwaukee, WI, USA) FloStent™ is a novel nitinol prostatic stent which is delivered using a flexible cystoscope in conjunction with either a custom deployment tool or a standard grasping forceps. Implant position can be adjusted using standard endoscopic equipment. The device can be explanted using standard grasping techniques via flexible or rigid cystoscopy.

In this study, we sought to determine the feasibility and safety of the FloStent™ implant in men with LUTS secondary to BPH.

## Materials and methods

**Fig. 1:**
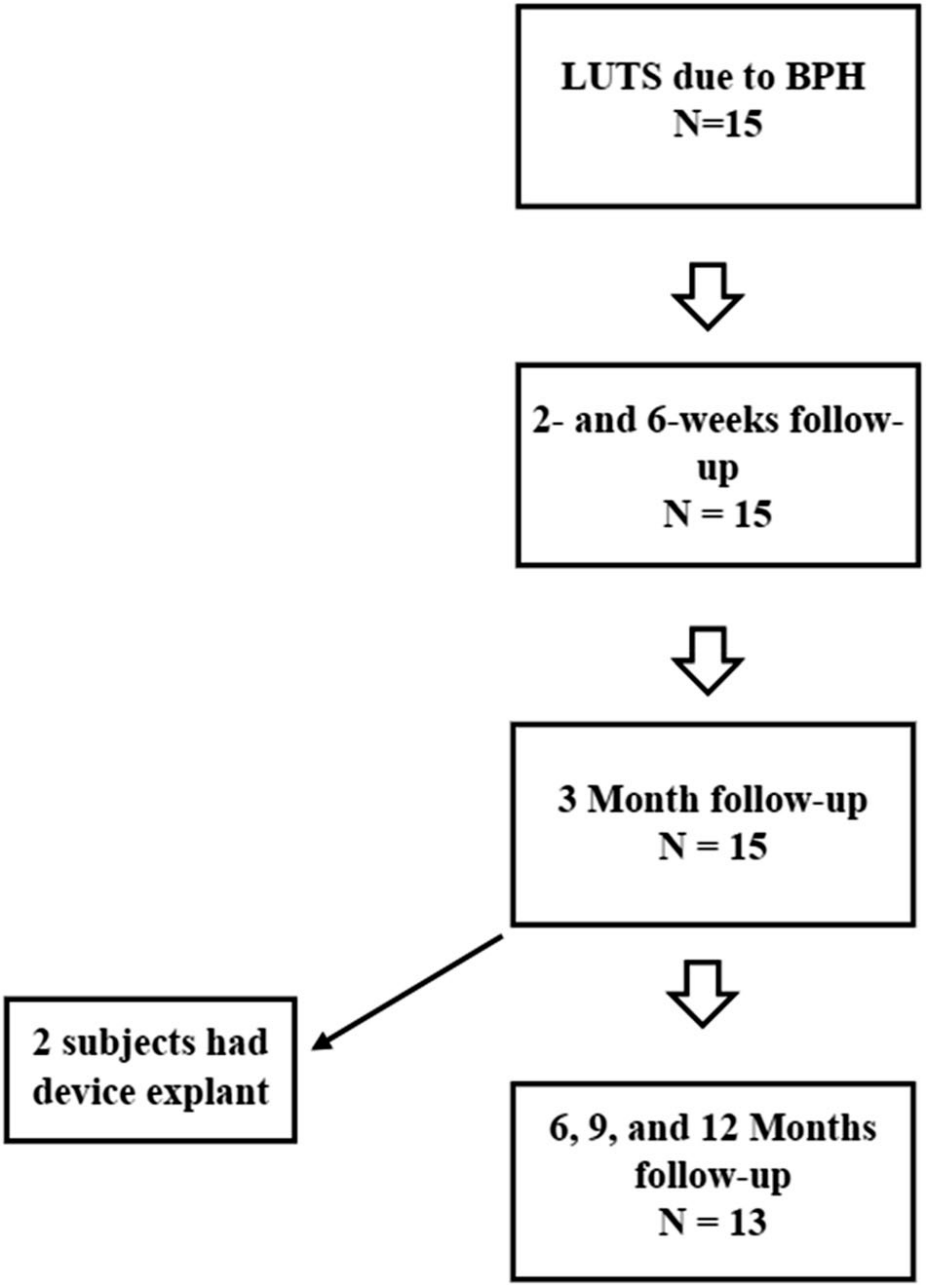
Study schematic.

This was a single-center, single-arm study to evaluate the technical feasibility and safety profile of the FloStent™ System in men ≥45 years of age with BPH. Descriptive statistics were employed to examine perioperative data, acute safety, and rapidity of symptom relief. The primary endpoints of the study were device implantability, device tolerability, and device retrievability. The original protocol called for a three-month study duration, with all subjects originally consenting to planned removal of the stent at the three-month timepoint. However, due to encouraging safety and efficacy data collected at the initial follow-up visits, the original protocol was amended to allow subjects to retain the stent for up to one year. All subjects were thus given the option to undergo device removal at three months, per the original protocol, or continue in the study and undergo device removal at 12 months, per the amended protocol (Fig. 1). Those subjects opting for study extension provided a separate informed consent for the study extension. Because the study included first-in-human device use, and all subjects were potential candidates for BPH intervention, all subjects were offered surgical standard-of-care therapy (TURP) after device explant, in order to ensure standard-of-care treatment was ultimately delivered. The protocol and all amendments (Rev 1.0, 1.1, 2.0, 2.1) were approved by local institutional review board (Comite de Bioetica de la Investigacion del Instituto Commemorativo Gorgas, CBI-ICGES) and national health ministry in Panama.

The study cohort was enrolled as a first-in-human (FIH) trial. The primary focus was implantability and safety. As such, participants were recruited based on either IPSS of >15 or peak urinary flow rate (Qmax) of ≤12 mL/s.

The formal inclusion criteria for this study encompassed men ≥40 years of age, with a prostate volume range of 25–80 cc, an International Prostate Symptom Score (IPSS) greater than 15 or a peak urinary flow rate (Qmax) of 12 mL/s or less, and a prostatic urethral length range of 2–4.5 cm (visualized via cystoscopy). Exclusion criteria included prostatic urethral length less than 2 cm, estimated prostatic volume less than 25cc or greater than 80cc, presence of an obstructing intravesical prostatic median lobe, urinary incontinence due to an incompetent external sphincter, and urethral pathologies preventing insertion of the delivery system, i.e. urethral stricture. Additional exclusion criteria included current symptomatic urinary tract infection or significant visible hematuria and subjects with a known allergy to nickel or titanium. Participants were also excluded if they had a known or suspected urological condition other than benign prostatic hyperplasia affecting voiding function, or neurogenic bladder and/or sphincter abnormalities.

Follow up assessments were performed at visits at 2, 6, 12, 26, 36, and 52 weeks. Though the study was not designed to make efficacy claims or test a hypothesis, clinical parameters including IPSS, Qmax, PVR, International Index of Erectile Function (IIEF), and Visual Analog Scale (VAS) were collected at baseline and at each follow up assessment. Additionally, the VAS was collected at the time of procedure.

### FloStent implantation

**Fig. 2:**
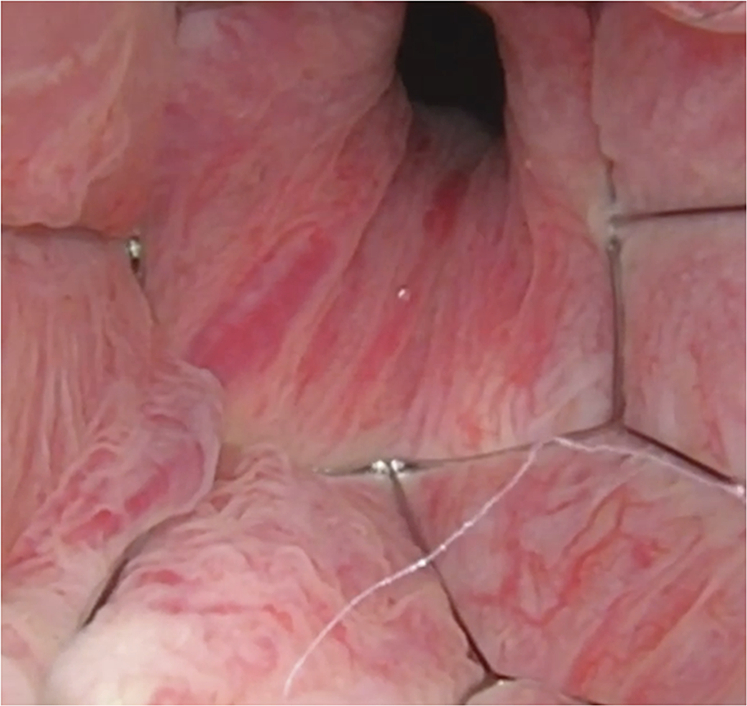
Endoscopic view of implanted FloStent.

The patient was placed in supine position and a transabdominal or transrectal ultrasound was conducted to confirm prostatic size. Perioperative antibiotics based on local antibiotogram and intravenous and/or local anesthesia (intra-urethral gel) were administered. A diagnostic flexible cystoscopy was performed to assess prostatic urethral anatomy. Prostatic urethral measurement was conducted using the cystoscopic pullback technique, measuring from the bladder neck to verumontanum, after which the cystoscope was withdrawn. Implant size was selected based on the measured prostatic anatomy. Once the cystoscope was within the prostatic urethra, the delivery tool was used to push the stent out of the cystoscope’s working channel. Once the stent was fully exposed and within proper position, the actuator on the grasper or delivery tool was used to release the implant. The delivery tool was then withdrawn. The cystoscope was used to assess implant patency and position. The stent when properly positioned was placed fully within the prostatic urethra, without crossing the bladder neck or external urinary sphincter (Fig. 2). If needed, stent position was adjusted using standard grasping forceps or by utilizing the tip of the flexible cystoscope. Once the implant position was visually satisfactory, the cystoscope was withdrawn. Voiding was confirmed via post void residual prior to postoperative discharge.

### FloStent retrieval

Device retrieval (explant) was safely performed in all patients. Two devices were removed per the original protocol at 12 weeks. Both patients declined TURP and were doing well at time of last clinical follow-up. 13 devices were removed at 52 weeks per the amended protocol (study extension). Twelve patients underwent TURP at the time of device retrieval, which was performed with a bipolar electrosurgical loop. In the remaining patient, TURP was deferred by the investigator due to a narrow penile urethra which would have required calibration with sounds; the patient reported good voiding after device removal and thus far has not pursued TURP.

Since this study represented the first experience with device retrieval in humans, retrievals were performed under general anesthesia in all patients. Two devices (both at 52 weeks) were removed with a flexible cystoscope; the remainder were removed with a rigid cystoscope. Standard graspers were used in all cases. No electrosurgical or laser energy was required for device retrieval. Minimal to mild mucosal bleeding was observed immediately post-explant.

In patients who did not undergo TURP at time of device explant, light cautery was used to control bleeding at the explant site. A postoperative catheter was left in place overnight in these patients.

## Results

Mean age of the cohort was 58.07 ± 9.56 years (range 42–74). Mean prostate volume was 44.8 cc (range 20–75). The PUL was 2.92 ± 0.95 cm (range 2–5.8). Baseline parameters appear in Table 1.

**Table 1:** Baseline characteristics.

Baseline characteristics – Arm 1 (LUTS due to BPH)	Results Mean ± SD [Min, Max] (*N* = 15)
**Age**, year	58.07 ± 9.56 [42, 74] (15)
**Prostate size**, mL	44.80 ± 14.35 [20, 75] (15)
**PUL**, cm	2.92 ± 0.95 [2, 5.8] (15)
**IPSS Total**, score	25.2 ± 5.5 [19, 33] (15)
**Qmax**, mL/Second	14.9 ± 9.1 [3, 38.9] (15)
**PVR**, mL	19.7 ± 20.4 [0, 60.0] (15)
**Cr** (Serum Creatinine), mg/dL	1.0 ± 0.4 [0.78, 2] (10)
**PSA**, ng/mL	1.8 ± 1.9 [0.47, 6.5] (11)
**VAS Pain Score**	0.13 ± 0.52 [0,2] (15)

All attempts at implantation were successful, using standard flexible cystoscopes. Both Olympus and Storz scopes were employed.

At the 3-month and 12-month follow-up, IPSS reduction was >30% in all subjects and average Qmax increase was >2 mL/sec across the cohort. No significant changes in PVR were noted.

A total of 13 AEs were reported and resolved, of these 8 were grade 1, 5 were grade 2, there were no grade 3,4, or 5 AEs. The most common AEs were related to discomfort with urination and/or ejaculation and were self-limited in nature.

## Discussion

The findings of this study demonstrate favorable technical feasibility and an excellent safety profile of the FloStent™ device, which is a novel minimally invasive surgical therapy (MIST) for men with benign prostatic hyperplasia (BPH) secondary to lower urinary tract symptoms (LUTS). FloStent™ offers a promising alternative to traditional BPH treatments, particularly for patients who prefer to avoid the long-term side effects associated with pharmacological treatments, and the irreversibility and sometimes prolonged recovery period associated with existing procedural therapies for BPH.

The primary findings of this first-in-human study are (a) that the FloStent can be implanted through the working channel of a standard flexible cystoscope, (b) the device is well-tolerated given that 13 of 15 patients opted to extend study participation and thus retained the stent for one year, and (c) the device is safely explantable using standard urological equipment up to one year after implantation.

First-in-human studies are intended to evaluate technical feasibility and safety. Device changes and modifications are often made based on study results. Therefore, definitive claims surrounding efficacy and clinical benefit cannot be made in such early studies. Due to these limitations, it is most appropriate to present first-in-human efficacy results in aggregate format, and this study’s aggregate results suggest a likely clinical benefit from the FloStent implant. The reported changes in IPSS and Qmax are considered clinically meaningful improvement (CMI), since the minimal clinically important difference (MCID) for IPSS reduction is >30% and Qmax improvement is 2 mL/sec. ^2^ It should be noted that Qmax eligibility for the study was not tightly restricted, and some patients with Qmax >12 mL/sec were implanted.

The significant reduction in IPSS at two weeks suggests that the recovery period for the FloStent may be shorter than that of BPH therapies that cut, pierce, dilate, or ablate tissue. Furthermore, IPSS reductions and Qmax improvements appear to be durable, meeting MCID criteria out to the 12-month timepoint.

While the study reported a total of 13 adverse events (AEs), all were resolved, and none resulted in serious complications. The majority of AEs were assessed as related to the study device, with a smaller number potentially related or not related. This suggests that while the FloStent™ is associated with some risk, the complications appear to be minimal and are likely outweighed by the benefits in symptom relief and improved urinary function.

As a feasibility study with a relatively small sample size (*n* = 15) and non-randomized design, the results should be interpreted with caution. The open-label nature of the study may introduce bias, and the absence of a control group limits the ability to attribute all observed effects solely to the FloStent™ device. The study was designed as a safety study and did not specify hypothesis testing for measured endpoints. It was therefore not powered to definitely establish clinical effectiveness. Additionally, the follow-up period, while extended to 12 months for some participants, does not capture long-term outcomes beyond one year, which would be necessary to fully assess the durability of the treatment. Nevertheless, the overall outcome profile provides an encouraging positive signal for the FloStent’s clinical effectiveness.

The FloStent is differentiated from other implantable BPH device therapies which are either irretrievable (e.g. UroLift) or specifically designed for short-term use and tissue remodeling (iTIND). The FloStent exerts therapeutic action while in place, offering near-immediate symptom relief without tissue trauma, but is designed for retrievability if clinically warranted, making it an ideal first-line option for those patients with BPH who prioritize ease of recovery and option preservation. Delivery via flexible cystoscope provides patient comfort, while universality (compatible with standard flexible cystoscopes) may reduce provider-related barriers to adoption.

## Conclusions

In summary, this study provides promising preliminary evidence that the FloStent™ device is a technically feasible and safe approach to BPH device therapy. Improvements in LUTS and uroflowmetry parameters suggest clinical benefit. This minimally invasive reversible therapy could offer a valuable treatment for patients seeking relief from BPH symptoms, without the risks associated with more invasive surgical procedures or the side effects of long-term medication use. While early results appear promising, it will be future studies with larger sample size and/or randomization that will ultimately validate the safety and efficacy profile of the FloStent System. Future investigations into comparison with medications and MISTs will be important to establish the role of FloStent™ in the broader BPH treatment landscape.

## Data Availability

The datasets generated during and/or analysed during the current study are available from the corresponding author on reasonable request.
